# Disparities in Receipt of Smoking Cessation Assistance Within the US

**DOI:** 10.1001/jamanetworkopen.2022.15681

**Published:** 2022-06-01

**Authors:** Kristin G. Maki, Robert J. Volk

**Affiliations:** 1 Department of Health Services Research, University of Texas MD Anderson Cancer Center, Houston

## Abstract

This cross-sectional study analyzes sociodemographic variations in receiving smoking cessation assistance from health professionals by individuals who reported smoking, or quitting, within the past year.

## Introduction

Although tobacco use has decreased in recent decades, there are disparities related to its use and receipt of cessation assistance.^1^ Our objective was to analyze sociodemographic variations in receiving cessation assistance from health professionals (HPs) by individuals who reported smoking, or quitting, within the past year.

## Methods

We used cross-sectional data from the 2020 National Health Interview Survey.^2,3^ Our study used a publicly available, anonymized database; thus, it was exempt from institutional review board oversight and the need for informed consent, in accordance with 45 CFR §46. This study follows the Strengthening the Reporting of Observational Studies in Epidemiology (STROBE) reporting guideline. For additional information on our methods, see the eAppendix and eTable in the Supplement.

Our outcome focused on the following item, “In the past 12 months, has a doctor, dentist, or other health professional advised you about ways to stop smoking or prescribed medication to help you quit?” Study interviewers asked this question of adults who saw an HP within the past year and reported currently, or quitting, smoking in the past year.

We also included sociodemographic characteristics and health-related indicators in our analysis. We included participants’ self-reported race and ethnicity because of these factors’ associations with smoking disparities.^1^

We used the survey package in RStudio statistical software version 1.4.1106 (R Project for Statistical Computing) to account for the complex sampling design and weighting. We used hierarchical multivariable logistic regression to assess the association between variables, with a significance level of *P* < .05 (2-sided). Data analysis was performed in February 2022.

## Results

Our sample (weighted number, 25 856 639 individuals; mean [SD] age, 48.38 [15.84] years; 12 849 072 female individuals) included individuals who had seen an HP and smoked currently, or quit, in the past year. There were higher odds of receiving cessation assistance for respondents who were aged 70 to 79 years compared with those younger than 30 years (odds ratio [OR], 2.18; 95% CI, 1.09-4.34), for respondents in medium-to-small metropolitan areas compared with nonmetropolitan regions (OR, 1.63; 95% CI, 1.17-2.29), for those who have 1 or more places to receive preventive care compared with those with no usual place (OR, 2.31; 95% CI, 1.35-4.03), for respondents who had a cessation attempt in the past year compared with those who did not (OR, 1.53; 95% CI, 1.16-2.00), and for those who had received a chronic obstructive pulmonary disease diagnosis compared to those who had not received a diagnosis (OR, 1.60; 95% CI, 1.15-2.25) (Table).

**Table.** Demographic, Access, and Smoking History Differences in Receipt of Tobacco Cessation Assistance

Variable	OR (95% CI)
Sociodemographic characteristics (pseudo *R*^2^ = 0.11)	
(Intercept)	0.37 (0.15-0.91)
Sex	
Male	1 [Reference]
Female	0.88 (0.68-1.13)
Age, y	
18-29	1 [Reference]
30-39	1.30 (0.76-2.12)
40-49	1.63 (0.92-2.90)
50-59	1.62 (0.90-2.91)
60-69	1.79 (1.00-3.23)
70-79	2.18 (1.09-4.34)
≥80	0.91 (0.37-2.24)
Race and ethnicity	
American Indian or Alaska Native	0.59 (0.35-0.99)
American Indian or Alaska Native, plus other group^a^	0.99 (0.78-1.82)
Hispanic or Latino	0.59 (0.42-2.30)
Non-Hispanic	
Asian	0.98 (0.43-2.24)
Black	1.19 (0.25-1.37)
White	1 [Reference]
Other or multiple^a^	2.93 (0.91-9.40)
Education	
Less than high school	1 [Reference]
High school or General Educational Development diploma	0.72 (0.48-1.08)
Less than 4-y college	0.91 (0.60-1.38)
4-y college	0.84 (0.50-1.41)
Graduate degree	1.04 (0.52-2.10)
Income poverty ratio	
<100%	1 [Reference]
100%-199%	0.98 (0.68-1.42)
≥200%	1.02 (0.72-1.44)
Marital status	
Not married	1 [Reference]
Married or living as married	1.02 (0.79-1.30)
Sexual orientation	
Straight	1 [Reference]
Gay or lesbian	1.96 (0.91-4.23)
Bisexual	1.41 (0.56-3.52)
Something else or do not know	0.86 (0.35-2.10)
Geographical factors (pseudo *R*^2^ = 0.13)	
Region	
Northeast	1 [Reference]
Midwest	0.72 (0.50-1.03)
South	0.65 (0.46-0.91)
West	0.66 (0.45-0.98)
Urbanicity	
Nonmetropolitan area	1 [Reference]
Medium and small metropolitan	1.63 (1.17-2.29)
Large fringe metropolitan	1.24 (0.87-1.78)
Large central metropolitan	1.26 (0.86-1.84)
Access to care (pseudo *R*^2^ = 0.15)	
Insurance type	
Private	1 [Reference]
All public	1.07 (0.79-1.47)
Other	1.38 (0.84-2.27)
Uninsured	0.60 (0.38-0.94)
Usual place to get preventive care	
No	1 [Reference]
Yes	2.31 (1.33-4.03)
Comorbidities (pseudo *R*^2^ = 0.17)	
Prior cancer diagnosis	
No	1 [Reference]
Yes	1.34 (0.93-1.94)
Diagnosis of chronic obstructive pulmonary disease, emphysema, or chronic bronchitis	
No	1 [Reference]
Yes	1.60 (1.15-2.25)
Smoking history (pseudo *R*^2^ = 0.16)	
Smoking cessation attempt in past 12 mo	
No	1 [Reference]
Yes	1.53 (1.16-2.00)
Smoking pack-years	
<20	1 [Reference]
20-29	1.33 (0.84-2.12)
≥30	1.22 (0.74-2.02)
Estimated eligibility for lung cancer screening^b^	
Not eligible	1 [Reference]
Eligible	1.12 (0.62-2.03)

Abbreviation: OR, odds ratio.

^a^ “The following four single-race categories are available for Sample Adult and Sample Children in the public-use files: (1) White; (2) Black or African American; (3) Asian; and (4) American Indian or Alaska Native (AIAN). The only multiple race category available in the public-use files is AIAN and another race. Sample Adult and Sample Child respondents indicating a single race other than the four mentioned or reporting more than one race, other than including AIAN, were combined into the ‘other single and multiple races’ category.”^3^

^b^ Eligibility was estimated using the 2021 USPSTF recommendation on lung cancer screening (the recommendation had not been updated at the time of data collection). Thus, individuals who were aged 50 to 80 years, with a smoking history of 20 or more pack-years, and currently smoking or quit within the past 15 years were considered eligible.

There were significantly lower odds of receiving cessation assistance for those in the Southern (OR, 0.65; 95% CI, 0.46-0.91) and Western regions (OR, 0.66; 95% CI, 0.45-0.98), compared with the Northeast, and for those without health insurance compared with those who have private insurance (OR, 0.60; 95% CI, 0.38-0.94). Results are summarized in the Table.

## Discussion

In this cross-sectional study, we found several disparities in receipt of cessation assistance reported by US adults who smoke, including region of the US, urban vs rural categorization, health insurance status, and having a usual place to receive preventive care. These findings highlight the pervasiveness of disparities associated with smoking cessation assistance based on sociodemographic variables.^1,4,5,6^

Although we saw an increased likelihood in receiving cessation assistance for respondents who had received a diagnosis of chronic obstructive pulmonary disease or similar conditions, our results did not show differences for those who had received a diagnosis of cancer. Similarly, there were no differences based on smoking pack-year history or estimated eligibility for lung cancer screening among those who currently smoke (pack-year information for former smoking is not available in this data set).

There were some limitations to this study. The data were collected as part of a national survey and may be prone to self-report bias. We also are not able to ascertain the type of HP who provided cessation assistance or its frequency. In addition, the data are cross-sectional and cannot be used to establish causal inferences.

Our study’s results highlight areas where sociodemographic gaps in receipt of smoking cessation assistance persist, including age, geographical region, and access to care. Future efforts should be focused to help mitigate tobacco-related disparities.

## Notes

Supplement. **eAppendix.** Supplemental Methods
eReferences
**eTable. **Variables Included in Analysis With Frequencies Reported
