# Supplementary material for: Disparities in Receipt of Smoking Cessation Assistance Within the US
Source: JAMA Netw Open. 2022 Jun 1;5(6):e2215681. doi: 10.1001/jamanetworkopen.2022.15681 (PMC9161016; doi:10.1001/jamanetworkopen.2022.15681)
Supplement: Supplement. — eAppendix. Supplemental Methods eReferences eTable. Variables Included in Analysis With Frequencies Reported [file jamanetwopen-e2215681-s001.pdf]

Supplemental Online Content

Maki KG, Volk RJ. Disparities in receipt of smoking cessation assistance within the US. *JAMA Netw Open*. 2022;5(6):e2215681. doi:10.1001/jamanetworkopen.2022.15681

eAppendix. Supplemental Methods

eReferences

eTable. Variables Included in Analysis With Frequencies Reported

This supplemental material has been provided by the authors to give readers additional information about their work.

© 2022 Maki KG et al. *JAMA Network Open.*

eAppendix. Supplemental Methods

Data Source

We used data from the public-use 2020 National Health Interview Survey[1], which is conducted by the National Center for Health Statistics within the Centers for Disease Control and Prevention (CDC). The data were collected with in-person interviews from January through March 2020 and shifted to telephone interviewing from March through June 2020. Data collection from July through December was completed primarily with telephone interviews with some in-person interviewing. Complete details about the data collection process are available in the 2020 National Health Interview Survey (NHIS) Survey Description, published by the National Center for Health Statistics in September 2021[2].

Measures

The outcome variable was derived from the following yes/no question, "In the past 12 months, has a doctor, dentist, or other health professional advised you about ways to stop smoking or prescribed medication to help you quit?" This question was asked to adults whose response to "About how long has it been since you last saw a doctor or other health professional about your health?" was "Within the past year (anytime less than 12 months ago)", and who reported smoking currently or quitting within the past year.

The independent variables included several sociodemographic items that have been shown to be related to tobacco use or disparities related to cessation assistance (see manuscript for references). Access to care was included with health insurance as well as having a usual place to receive preventive care. Smoking history was also assessed.

Analysis Plan

Our sample (weighted *N* = 25,856,639) included adults who met the following criteria: 1) reported seeing a health professional within the past 12 months, and 2) reported currently smoking or quitting within the past 12 months. Quantitative variables that used groupings we computed (e.g., participant age groups and smoking pack-years) allowed us to assess

© 2022 Maki KG et al. *JAMA Network Open.*

differences between potential risk factors for potential health outcomes related to smoking (e.g., lung cancer).

We assessed potential bias due to missing data. None of variables had more than 5% of cases missing; we used listwise deletion for the analysis. We used the following packages within RStudio[3] for this study: haven[4], jtools[5], survey[6], finalfit[7]. We conducted our main analysis with the survey[6] package. This allowed us to account for the complex sampling design and use the weights provided with the NHIS[1] data to represent the U.S. population at the time of data collection. In our analysis, we used hierarchical multivariable logistic regression with 5 blocks to control for potentially confounding variables.

eReferences

1. National Center for Health Statistics. National Health Interview Survey, 2020. Public-use data file and documentation. https://www.cdc.gov/nchs/nhis/data-questionnaires-documentation.htm. 2021
2. National Center for Health Statistics. Survey Description, National Health Interview Survey, 2020. https://ftp.cdc.gov/pub/Health_Statistics/NCHS/Dataset_Documentation/NHIS/2020/srvydesc-508.pdf
3. R Core Team (2021). R: A language and environment for statistical computing. R Foundation for Statistical Computing, Vienna, Austria. URL https://www.R-project.org/.
4. Hadley Wickham and Evan Miller (2021). haven: Import and Export 'SPSS', 'Stata' and 'SAS' Files. R package version 2.4.3. https://CRAN.R-project.org/package=haven
5. Long JA (2020). _jtools: Analysis and Presentation of Social Scientific Data_. R package version 2.1.0, <URL: https://cran.r-project.org/package=jtools>.
6. Lumley (2020) "survey: analysis of complex survey samples". R package version 4.0.
7. Ewen Harrison, Tom Drake and Riinu Ots (2021). finalfit: Quickly Create Elegant Regression Results Tables and Plots when Modelling. R package version 1.0.4. https://CRAN.R-project.org/package=finalfit

© 2022 Maki KG et al. *JAMA Network Open.*

**eTable.** Variables Included in Analysis With Frequencies Reported			
**Variable**	**Categories**	**Full Sample, weighted n (%)**	**Only SMKTLK_A = yes, weighted n (%)**
Outcome variable			
SMKTLK_A (In the past 12 months has a doctor, dentist, or other health professional advised you about ways to stop smoking or prescribed medication to help you quit?	Yes	13,393,474 (51.8%)	13,393,474 (100%)
	No	12,463,165 (48.2%)	NA
Independent variables			
SEX_A (Sex of sample adult)	Male	13,103,183 (50.5%)	6,680,357 (49.9%)
	Female	12,849,072 (49.5%)	6,713,117 (50.1%)
AgeDecades (recode of AGEP_A)	18-29	3,755,694 (14.5%)	1,261,349 (9.4%)
	30-39	4,887,138 (18.8%)	2,059,229 (15.4%)
	40-49	4,680,841 (18.1%)	2,284,015 (17.1%)
	50-59	5,296,646 (20.4%)	3,014,459 (22.5%)
	60-69	4,797,787 (18.5%)	3,129,682 (23.4%)
	70-79	2,106,657 (8.1%)	1,457,190 (10.9%)
	80+	404,955 (1.6%)	172,824 (1.3%)
HISPALLP_A (single and multiple race groups with Hispanic origin)	Hispanic	2,750,394 (10.6%)	907,689 (6.8%)
	NH White	17,494,545 (67.4%)	9,459,851 (70.6%)
	NH Black/African American	3,619,440 (13.9%)	1,960,013 (14.6%)
	NH Asian	988,465 (3.8%)	418,016 (3.1%)

© 2022 Maki KG et al. *JAMA Network Open.*

	NH AIAN	393,882 (1.5%)	185,354 (1.4%)
	NH AIAN and any other group	313,842 (1.2%)	170,690 (1.3%)
	Other single and multiple races	391,688 (1.5%)	291,860 (2.2%)
Education levels (recode of EDUC_A)	Less than high school/GED	4,608,403 (18.0%)	2,558,318 (19.4%)
	High school/GED	10,041,044 (39.2%)	5,161,531 (39.0%)
	Less than 4-year college	7,876,289 (30.7%)	4,043,798 (30.6%)
	4-year college	2,235,716 (8.7%)	1,032,191 (7.8%)
	Graduate degree	880,997 (3.4%)	423,846 (3.2%)
Income to poverty ratios (recode of RATCAT_A, which used published poverty thresholds for the previous calendar year in the calculations)	Less than 100%	15,068,820 (58.1%)	7,524,100 (56.2%)
	100-199%	4,662,862 (18.0%)	2,571,556 (19.2%)
	200% and above	6,220,573 (24.0%)	3,297,817 (24.6%)
Married (recode of MARITAL_A)	Neither	11,738,503 (45.9%)	6,072,802 (46.1%)
	Married or living with a partner	13,837,272 (54.1%)	7,113,625 (53.9%)
Sexual orientation (recode of ORIENT_A)	Straight	23,862,957 (93.6%)	12,256,387 (93.5%)
	Gay/lesbian	580,875 (2.3%)	386,726 (3.0%)
	Bisexual	669,625 (2.6%)	311,494 (2.4%)
	Something else or don’t know	383,123 (1.5%)	150,205 (1.1%)
Household region (REGION)	Northeast	4,110,062 (15.8%)	2,446,763 (18.3%)
	Midwest	6,455,421 (24.9%)	3,442,523 (25.7%)

© 2022 Maki KG et al. *JAMA Network Open.*

	South	11,002,402<br><br>(42.4%)	5,338,338 (39.9%)
	West	4,384,369 (16.9%)	2,165,850 (16.2%)
URBBRL (2013 NCHS Urban-Rural Classification Scheme for Counties)	Nonmetropolitan	5,533,749 (21.3%)	2,718,325 (20.3%)
	Medium and small metro	8,374,707 (32.3%)	4,744,848 (35.4%)
	Large fringe metro	5,646,371 (21.8%)	2,759,785 (20.6%)
	Large central metro	6,397,428 (24.7%)	3,170,516 (23.7%)
Insurance type (recode of COVER65_A and COVER_A to combine all age groups)	Private	12,280,917<br><br>(47.4%)	6,031,978 (45.1%)
	All public	8,248,969 (31.9%)	4,901,947 (36.7%)
	Other types	2,070,127 (8.0%)	1,375,209 (10.3%)
	Uninsured	3,285,565 (12.7%)	1,059,212 (7.9%)
Usual place for preventive care (recode of USUALPL_A to make binary variable)	No usual place(s) to receive preventive care	2,171,769 (8.4%)	616,379 (4.6%)
	One or more place(s) to receive preventive care	23,780,486<br><br>(91.6%)	12,777,094 (95.4%)
Cancer diagnosis (CANEV_A)	Yes	2,537,880 (9.8%)	1,665,433 (12.4%)
	No	23,399,016<br><br>(90.2%)	11,723,238 (87.6%)
COPD, emphysema, or chronic bronchitis diagnosis (COPDEV_A)	Yes	3,883,512 (15.0%)	2,843,467 (21.2%)
	No	22,016,011<br><br>(85.0%)	10,540,140 (78.8%)
During the past 12 months, have you stopped smoking for more than one day because you were trying to	Yes	11,731,247<br><br>(50.1%)	6,465,138 (52.7%)

© 2022 Maki KG et al. *JAMA Network Open.*

quit smoking? (SMKQT12M_A)[a]	No	11,679,942 (49.9%)	5,809,837 (47.3%)
Pack year groups[b]	≤19	8,875,660 (52.2%)	4,544,112 (45.9%)
	20-29	3,209,042 (18.9%)	2,101,881 (21.3%)
	≥30	4,917,407 (28.9%)	3,244,628 (32.8%)
Eligible for LCS, using USPSTF 2021 recommendation[c]	Not eligible	19,783,957 (76.2%)	9,154,796 (68.4%)
	Eligible	6,168,298 (23.8%)	4,238,678 (31.6%)

[a]This item was only asked to participants who reported currently smoking.
[b]This was calculated with the following variables: YrsSmk = AGEP_A-SMKAGE_A; PacksDay = CIGNOW_A/20; PYs = YrsSmk*PacksDay; PYGroups=(Lowest thru 19=1) (20 thru 29=2) (30 thru Highest=3). The relevant variables only used data from participants who reported ever smoking 100 cigarettes in their lifetime.
[c]This was calculated with the following variables: Elig2021_computed=Age50_80 + PYs20plus where Age50_80=50-80 years = 1, else=0 and PYs20plus=20 or more pack-years = 1, <20=0.

© 2022 Maki KG et al. *JAMA Network Open.*
